# GH Overexpression Alters Spermatic Cells MicroRNAome Profile in Transgenic Zebrafish

**DOI:** 10.3389/fgene.2021.704778

**Published:** 2021-09-08

**Authors:** William B. Domingues, Tony L. R. Silveira, Leandro S. Nunes, Eduardo B. Blodorn, Augusto Schneider, Carine D. Corcine, Antônio S. Varela Junior, Izani B. Acosta, Mateus T. Kütter, Gonzalo Greif, Carlos Robello, Danillo Pinhal, Luís F. Marins, Vinicius F. Campos

**Affiliations:** ^1^Laboratório de Genômica Estrutural, Programa de Pós-Graduação em Biotecnologia, Centro de Desenvolvimento Tecnológico, Universidade Federal de Pelotas, Pelotas, Brazil; ^2^Laboratório de Biologia Molecular, Instituto de Ciências Biológicas, Universidade Federal do Rio Grande, Rio Grande, Brazil; ^3^Faculdade de Nutrição, Universidade Federal de Pelotas, Pelotas, Brazil; ^4^ReproPel, Programa de Pós-Graduação em Veterinária, Faculdade de Veterinária, Universidade Federal de Pelotas, Pelotas, Brazil; ^5^Unidad de Biología Molecular, Institut Pasteur, Montevideo, Uruguay; ^6^Laboratório Genômica e Evolução Molecular Departamento de Genética, Instituto de Biociências de Botucatu Universidade Estadual Paulista (UNESP), Botucatu, Brazil

**Keywords:** non-coding RNAs, epigenetic, *Danio rerio*, miRNA-seq, sperm motility, transgenic fish

## Abstract

Overexpression of growth hormone (GH) in *gh*-transgenic zebrafish of a highly studied lineage F0104 has earlier been reported to cause increased muscle growth. In addition to this, GH affects a broad range of cellular processes in transgenic fish, such as morphology, physiology, and behavior. Reports show changes such as decreased sperm quality and reduced reproductive performance in transgenic males. It is hypothesized that microRNAs are directly involved in the regulation of fertility potential during spermatogenesis. The primary aim of our study was to verify whether *gh* overexpression disturbs the sperm miRNA profile and influences the sperm quality in transgenic zebrafish. We report a significant increase in body weight of *gh*-transgenic males along with associated reduced sperm motility and other kinetic parameters in comparison to the non-transgenic group. MicroRNA transcriptome sequencing of *gh*-transgenic zebrafish sperms revealed expressions of 186 miRNAs, among which six miRNA were up-regulated (miR-146b, miR-200a-5p, miR-146a, miR-726, miR-184, and miR-738) and sixteen were down-regulated (miR-19d-3p, miR-126a-5p, miR-126b-5p, miR-22a-5p, miR-16c-5p, miR-20a-5p, miR-126b-3p, miR-107a-3p, miR-93, miR-2189, miR-202–5p, miR-221–3p, miR-125a, miR-125b-5p, miR-126a-3p, and miR-30c-5p) in comparison to non-transgenic zebrafish. Some of the dysregulated miRNAs were previously reported to be related to abnormalities in sperm quality and reduced reproduction ability in other species. In this study, an average of 134 differentially expressed miRNAs-targeted genes were predicted using the *in silico* approach. Kyoto Encyclopedia of Genes and Genomes (KEGG) pathway enrichment analysis demonstrated that the genes of affected pathways were primarily related to spermatogenesis, sperm motility, and cell apoptosis. Our results suggested that excess GH caused a detrimental effect on sperm microRNAome, consequently reducing the sperm quality and reproductive potential of zebrafish males.

## Introduction

Since the first proposal to use zebrafish (*Danio rerio*) in research (Creaser, 1934), the species have attained global popularity as an experimental model organism (Streisinger et al., 1981), and its use in science has grown and continues to grow rapidly (Kinth et al., 2013; Teame et al., 2019). Approximately 32 wild strains, such as AB, Tübingen long-fin, and Tüpfel long-Fin, are currently used (which excludes the local pet shop variants) in various studies (van den Bos et al., 2020; Silveira et al., 2021).^1^ These reports show more than 115,000 genetic alterations done in zebrafish, of which more than 47,000 are transgenic insertions (Ruzicka et al., 2019). The first transgenic fish, the F0104 strain, was developed in Brazil in 2004 (Figueiredo et al., 2007a). Through transgenesis, the gene sequences can be manipulated to assign characteristics of interest. In addition, animal cells can be artificially labeled for easy visualization (Ingham, 2009).

The F0104 strain zebrafish model used in this study carried two transgenes: (1) the β-actin promoter from carp (*Cyprinus carpio*) that drives the expression of coding DNA sequence (CDS) of growth hormone gene (*gh*) from the marine silverside *Odonthestes argentinensis* and (2) green fluorescent protein (GFP) from the jellyfish *Aequorea victoria* used as a transgenesis label controlled by the same promoter. In related studies, these transgenic zebrafish showed to have increased growth (Figueiredo et al., 2007b); better oxygen consumption (Rosa et al., 2008; Almeida et al., 2013a); Reactive oxygen species (ROS) production (Rosa et al., 2008); cognition (Studzinski et al., 2015); and regeneration capacity (Nornberg et al., 2016). However, on the other side, these strains have also shown to develop osmotic and energy imbalance (Almeida et al., 2013b); weakened immunity (Batista et al., 2014); deficits in antioxidant defense mechanism (da Rosa et al., 2011); less apparent sexual dimorphism (Figueiredo et al., 2007a); decrease in spermatic parameters and reproductive capacity of males (Figueiredo et al., 2013).

In the male reproductive system, growth hormone (GH) is said to interfere with testicular development and stimulate spermatogenesis by acting both directly and indirectly on testes through potentiation of gonadotropin, affecting the gonadal development by stimulating the expression of insulin-like growth factors (IGFs) (Gac et al., 1993; Berishvili et al., 2006; Miura et al., 2011). In fact, in both cases, GH may be considered as a co-factor for gonadotropin (Hull and Harvey, 2002) along with IGFs to specifically regulate the gonadal function (Gac et al., 1993). Overexpression of *gh* in other transgenic fish strains culminates in reproductive deficits, such as in tilapia (Rahman et al., 1998); Atlantic salmon (Moreau et al., 2011); coho salmon (Devlin et al., 2004); mud loach (Nam et al., 2002); and common carp (Cao et al., 2014). Some authors highlight that the factors that link somatic growth with reproductive deficits in *gh*-transgenic fish still need to be elucidated (Chen et al., 2018). A modern tool available for this task is the analysis of differential expression of microRNAs (miRNAs).

MicroRNAs are a class of endogenous non-coding RNAs (ncRNA), generally about 22 nucleotides in length regulating gene expressions canonically at the post-transcriptional level (Blödorn et al., 2021). To date, records in miRBase (Kozomara et al., 2019) and MiRGeneDB databases (Fromm et al., 2020) report 373 and 390 mature miRNA molecules are being expressed in zebrafish, respectively. Estimates suggest that between 30 and 60% of the total gene expressions can be regulated by miRNAs (Lewis et al., 2005; Xie et al., 2005; Friedman et al., 2008). MiRNAs are involved in virtually all biological processes in the cells of metazoans, including the spermatogenesis and formation of spermatozoa in zebrafish (Kotaja, 2014; Jia et al., 2015). Due to its wide distribution among species and role in regulating gene expressions, miRNAs have been used as biomarkers for various applications, including infertility diagnosis (Wang et al., 2015; Corral-vazquez et al., 2019; dos Santos da Silva et al., 2021).

Despite high maintenance of the F0104 strain being a major issue, the inherent reproductive deficit presented in the F0104 males provides an opportunity to understand the effects of *gh* expression levels on the reproduction potential using advanced approaches. Therefore, the F0104 strain has been used as an interesting model for investigations on spermatic quality, reproductive potency, and their determining factors in males. In addition, zebrafish is a translational model, and therefore the results obtained in this species can be extrapolated to other species, including humans (Vargas, 2018). Thus, the present study aimed to evaluate the sperm kinetic parameters as well as the sperm quality by sequencing, identifying, and quantifying miRNAs in sperm cells of *gh*-transgenic (*gh*+) and non-transgenic (NT) zebrafish, further elucidating the specific pathways influenced by transgenesis and lastly, to identify prospective candidate miRNAs as epigenetic biomarkers for the reproductive deficit of zebrafish males.

## Materials and Methods

### Zebrafish

The F0104 zebrafish strain used in the study were *gh*-transgenic males. The NT fish were siblings of the *gh* + zebrafish (having no genetic construct incorporated into its genome). Both transgenic and NT zebrafish were selected in their larval stage, and 20 fish from each type were distributed into four different aquaria (2 for *gh* + and 2 for NT) kept in a closed water recirculation system. Each aquarium was maintained in 15 L water with oxygen levels near saturation (>6 mg/L), pH close to 7.2, photoperiod 14:10 h light: dark cycle, and temperature of 28°C. Ammonia (NH_3_) and nitrite (NO_2_^–^) concentrations were measured three times a week using commercial kits (LabconTest Toxic Ammonia Freshwater; LabconTest Nitrite, Alcon^®^, Brazil) and corrected with partial water change when necessary. The levels of ammonia and nitrites showed to maintain below 0.011 and 0.25 mg/L, respectively. The fish were fed *ad libitum* twice a day with commercial fish food (ColorBits, Tetra^®^, Germany).

### Testes Excision

When the fish reached 15 months of age, which is normally considered adult age, presenting appropriate sperm production with normal sperm concentration and motility values (Johnson et al., 2018), 10 males from each experimental group were randomly captured by the same experienced operator (5 fish from each aquarium) and euthanized by hypothermic shock (in water with 4°C) for the removal of testes. One collected testis from each fish was placed in 100 μL of Beltsville Thawing Solution (BTS) (Pursel and Johnson, 1975) for semen analyses and the other preserved in TRIzol Reagent (Invitrogen, United States) for miRNA sequencing. This study was conducted in compliance with institutional, national, and international guidelines for using animals, and all the protocols used were performed by the guidelines and approved by the Ethics Committee of the Federal University of Rio Grande (FURG), Brazil, under the code 23116.008403/2018–32.

### Sperm Kinetics Parameters

Sperm cells were assessed by Computer Assisted Sperm Analysis (CASA) system (SpermVision^®^, Minitube, Germany) coupled to an optical microscope (Axio Scope A1^®^, Zeiss, Germany) and observed at 200 X magnification according to the method described by Acosta et al. (2016). To activate sperm cells, activation solution consisting of 119 mM sodium bicarbonate solution (NaHCO_3_) was prepared, added to semen in a 1:5 ratio, and plated on a slide. Variables assessed by CASA were: Total motility (TMO), Progressive motility (PMO), Velocity average path (VAP), Velocity curved line (VCL), Velocity straight line (VSL), and Linearity (LIN). For each sample, at least 500 cells were observed in 10 different fields.

The duration of sperm motility (in seconds) was evaluated using semen sample aliquots in 1:5 ratio using activation solution (prepared as earlier) and loaded on slides covered with a coverslip, a timer set, and time noted until sperm cells movement ceased.

### RNA Isolation From Sperm Cells

Total RNA was isolated from sperm cells using a method that combined TRIzol reagent (Invitrogen, United States) and RNeasy mini spin column (RNeasy Mini Kit, Qiagen Inc., Valencia, CA, United States), according to the manufacturer’s instructions with modifications. Briefly, testes samples were placed in 1 mL lysis solution containing 0.5% Triton and 0.1% SDS at room temperature for 30 min for removal of the contaminating somatic cells. The digested tissue solution was centrifuged at 50 × g for 5 min, and tissue debris in the pellet was discarded. To the supernatant, about 500 μL TRIzol reagent was added and thoroughly mixed. A known volume of chloroform was added to the homogenate, mixed vigorously, and incubated for 10 min at room temperature. The mixture was then centrifuged at 12,000 × g at 4°C for 15 min. After centrifugation, the upper aqueous phase was collected and mixed with an equal volume of 100% ethanol. The entire mixture was transferred to the RNeasy mini spin column. The RNA bound to the membrane of the spin column when centrifuged was subsequently removed using buffer RWT and buffer RPE and eluted in 30 μL RNase-free water. Concentration and purity of the isolated sperm RNA were measured using Agilent 4,200 TapeStation system and the Agilent RNA ScreenTape assay (Agilent Technologies, Santa Clara, United States) (Supplementary Figure 1). All RNA samples were stored at −80°C until used.

### MicroRNA Sequencing

Small RNA libraries of sperm cells were generated using Next Small RNA Library Prep Set (New England Biolabs, Inc., United States) for the Illumina platform. To prepare each library set, three sperm RNA samples in equal concentrations were pooled together. A total of eighteen samples were used for the small RNA library preparation process, which culminated in 3 libraries for the *gh* + group and 3 libraries for the NT group. The manufacturer’s instructions were followed throughout for the PCR amplification (15 cycles), taking 1,000 ng template as starting input. Following PCR amplification, the libraries were run in a 6% polyacrylamide gel, and the ∼140 bp bands were excised from the gels using a razor blade and quantified by BioAnalyzer 2,100 (Agilent Technologies, United States).

The concentrations of the six RNA libraries (3 from *gh* + and 3 from NT groups) were determined using the Invitrogen Qubit^TM^ dsDNA High Sensitivity Assay Kit. The libraries were pooled at equimolar concentrations before MiSeq (Illumina, United States) sequencing in 50-bp single-end reads configuration using v3 sequencing chemistry.

### MicroRNA Detection and Differential Expression Analysis

Raw sequence data were subjected to a cleaning process to remove 5′ and 3′ primer contaminants, N adaptors, polyA adaptors, sequences without index sequence tag, and adaptors shorter than 17 nucleotides. The alignment of sequences from these libraries and identification of miRNAs was performed using the webserver RNA toolbox,^2^ according to methods used by Schneider et al. (2018). Differential expression of miRNAs was analyzed using the package edgeR (version 3.34) for both the *gh* + and NT experimental groups. Those miRNAs with False Discovery Rate (FDR) > 0.05 and Fold Changes (FC) in expression > 2.0 were considered to be upregulated, while those with FDR < 0.05 and FC < 0.50 were considered to be downregulated.

### Target Prediction and Gene Ontology Enrichment Analysis

The tool mirPath version 3.0 (Vlachos et al., 2015) was used to identify genes regulated by differentially expressed miRNAs, using the database microT-CDS v.5.0 (Paraskevopoulou et al., 2013). The gene ontology (GO) terms of the most enriched miRNA targets were annotated using the same mirPath tool. The gene targets of miRNAs were organized based on their functional role in different biological processes. The KEGG molecular pathways regulated by the differentially expressed miRNAs (DEmiRNAs) with a *p*-value < 0.05 were considered as significantly enriched in our analysis.

### Statistical Analysis

The statistical analyses of sperm kinetics parameters were performed using Statistix version 10.0. Compiled datasets were evaluated by Student’s *t*-test, and the differences considered significant when *p* < 0.05. Data normality and homogeneity of variances were previously verified.

## Results

### Effects on Body Weight and Spermatozoa Kinetic Parameters

**FIGURE 1:**
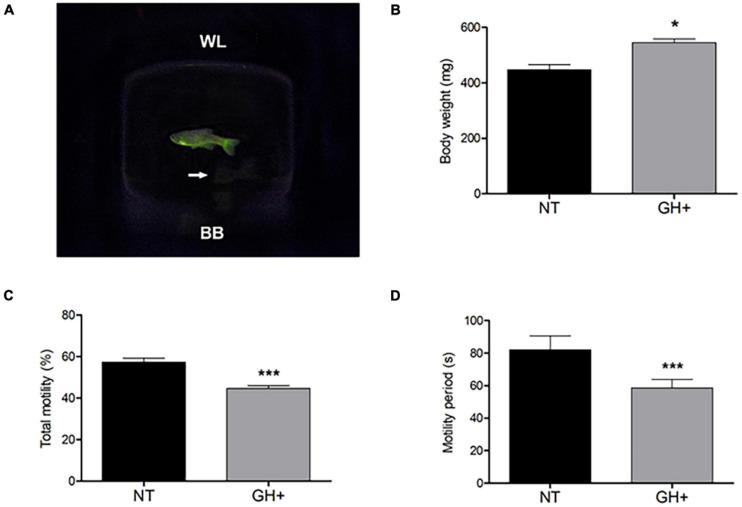
Indications of transgenic phenotype in a photographic image of male zebrafish, changes in body weight, and sperm kinetic parameters of *gh*-transgenic F0104 strain zebrafish (*Danio rerio*) and non-transgenic strain. **(A)** *gh*-transgenic zebrafish male from F0104 strain showing the ubiquitous and constitutive distribution of GFP expression; non-transgenic zebrafish shown in white arrows; **(B)** bodyweight characteristics; **(C)** total motile sperm; **(D)** sperm motility period. All *p*-values < 0.05 are summarized with one asterisk and < 0.0001 with three asterisks. Results are statistically expressed by mean ± standard error of the mean (SEM) (*n* = 10 for each experimental group). NT, non-transgenic; GH+, *gh*-transgenic; WL, water line; BB, bottom of beaker.

The biometric measure of weight (Figure 1B) demonstrated that transgenesis (Figure 1A) led to a significant increase in body weight of *gh* + individuals when compared to NT individuals (544.6 ± 13.59 g and 466.6 ± 19.13 g, respectively).

The results obtained through the CASA to evaluate sperm motility demonstrated that *gh* + individuals had low total motility (Figure 1C) and lesser duration of spermatozoa motility (Figure 1D) when compared to the NT individuals (*P* < 0.0001). In addition, a significant decrease in other kinetic parameters was observed, such as reduced progressive motility and lower distances covered, both of which relate to the fertilizing potential of sperm in these transgenic individuals (Table 1).

**TABLE 1:** Evaluation of sperm sample parameters in F0104 *gh*-transgenic and non-transgenic zebrafish (*Danio rerio*) by Computer Assisted Sperm Analysis (CASA) system: Progressive motility (PROG), Distance curved line (DCL), Distance average path (DAP), Distance straight line (DSL), Curvilinear velocity (VCL), Average path velocity (VAP), Straight-line velocity (VSL), Linearity of the curvilinear trajectory (LIN), Straightness (STR), the wobble of the curvilinear trajectory (WOB), the amplitude of lateral head displacement (ALH), and beat cross frequency (BCF).

Group	PROG (%)	DCL (μm)	DAP (μm)	DSL (μm)	VCL (μm/s)	VAP (μm/s)	VSL (μm/s)	LIN (%)	STR (%)	WOB (%)	ALH (μm)	BCF (Hz)
NT	51.43 ± 1.96	31.60 ± 0.69	29.24 ± 0.64	26.53 ± 0.65	67.70 ± 1.54	61.32 ± 1.56	55.91 ± 1.55	0.81 ± 0.004	0.91 ± 0.004	0.89 ± 0.005	1.01 ± 0.03	30.11 ± 0.38
GH +	38.11 ± 1.40	28.20 ± 1.02	25.72 ± 0.97	22.69 ± 0.99	59.26 ± 2.07	54.63 ± 2.15	47.88 ± 1.98	0.79 ± 0.004	0.88 ± 0.004	0.89 ± 0.005	0.89 ± 0.01	28.46 ± 0.60
*P* < 0.05	*	*	*	*							*	*

*Asterisk indicates a significant difference between groups (p < 0.05). Data are expressed as means ± standard error of the mean (SEM) (n = 10 for each experimental group). NT, non-transgenic; gh+, gh-transgenic.*

### Differential Expression of miRNAs in *gh-*Transgenic Strain

The microRNAome sequencing generated 21,981,994 raw reads from the six miRNA libraries altogether. After clean-up, a total of 186 known mature miRNAs were identified. Expression analysis of these miRNAs revealed 22 differentially expressed miRNAs (DEmiRNAs) in the *gh* + sperm cells (Table 2), including 16 down-regulated and 6 upregulated miRNAs in comparison to NT cells (Supplementary Table 1). Interestingly, for the miR-126 paralogs, both arms were down-regulated, suggesting that their 5p and 3p mature transcripts are functionally associated, controlling distinct genes that take part in the same gene regulatory network.

**TABLE 2:** List of differentially expressed microRNAs (miRNAs) in sperm cells of *gh*-transgenic and non-transgenic zebrafish.

	miRNAs	Relative expression	Adjusted *P*-value	#Target genes
Down-regulated in *gh*+	dre-miR-19d-3p	0.11	0.002	202
	dre-miR-126a-5p	0.13	0.0005	392
	dre-miR-126b-5p	0.13	0.0005	392
	dre-miR-22a-5p	0.15	0.0001	46
	dre-miR-16c-5p	0.16	0.0002	187
	dre-miR-20a-5p	0.18	0.0005	404
	dre-miR-126b-3p	0.20	0.001	6
	dre-miR-107a-3p	0.20	0.001	106
	dre-miR-93	0.21	0.001	371
	dre-miR-2189	0.26	0.0003	142
	dre-miR-202-5p	0.27	0.00001	16
	dre-miR-221-3p	0.27	0.0002	69
	dre-miR-125a	0.30	0.002	49
	dre-miR-125b-5p	0.31	0.0003	51
	dre-miR-126a-3p	0.42	0.002	6
	dre-miR-30c-5p	0.43	0.004	374
Up-regulated in *gh*+	dre-miR-146b	4.99	0.000001	30
	dre-miR-200a-5p	6.38	0.003	26
	dre-miR-146a	6.90	0.000001	20
	dre-miR-726	8.31	0.0008	47
	dre-miR-184	17.44	0.00008	13
	dre-miR-738	70.59	0.000004	1

### Molecular Pathways Regulated by miRNAs

An average of 134 putative target genes for the 22 DEmiRNAs was predicted using microT-CDS database (Table 2). After enrichment analysis, nine KEGG pathways were identified to be significantly related to DEmiRNAs gene targets (Table 3). Accordingly, down-regulated miRNAs were shown to be enriched for the FoxO signaling pathway, which plays a role in protein processing in the endoplasmic reticulum, sulfur relay system, apoptosis, and associated with the p53 signaling pathway. On the other hand, the upregulated miRNAs were shown to be enriched for pathways involved in mucin-type O-Glycan biosynthesis, glycosaminoglycan biosynthesis-heparan sulfate/heparin, N-Glycan biosynthesis, Toll-like receptor signaling, and glycerophospholipid metabolism. All these observations together indicate that the GH overexpression affects gene expression of key sperm motility pathways by altering the levels of regulatory miRNAs.

**TABLE 3:** KEGG molecular pathways differentially expressed miRNA-targeted genes in sperm cells of *gh*-transgenic and non-transgenic zebrafish.

	KEGG molecular pathways	*P*-value	Target genes	#miRNA
Down-regulated miRNAs	FoxO signaling pathway	0.0006	26	11
	Protein processing in endoplasmic reticulum	0.004	22	9
	Apoptosis	0.01	14	8
	*p53* signaling pathway	0.04	11	8
Up-regulated MiRNAs	Mucin type O-Glycan biosynthesis	0.00001	1	2
	Glycosaminoglycan biosynthesis	0.00002	1	1
	N-Glycan biosynthesis	0.002	1	1
	Toll-like receptor signaling pathway	0.01	2	2
	Glycerophospholipid metabolism	0.04	1	1

## Discussion

To the best of our knowledge, the present study is the first to demonstrate the effects of GH overexpression on the microRNAome profile of sperm cells in *gh* + transgenic zebrafish. We demonstrated that 16 miRNAs were down-regulated, and 6 miRNAs were upregulated in *gh* + transgenic fish when compared to the NT fish. Some of these candidates’ differentially expressed miRNAs have been previously reported to play a potential role in influencing spermatic quality and reproductive success. Furthermore, a high level of sequence conservation in the aforementioned miRNAs of the metazoan genome helps up to directly compare the current findings in zebrafish (*Danio rerio*) with known findings of other species.

Among the down-regulated miRNAs identified, miR-20a-5p was earlier reported to express at lower levels in bovine sperm (Capra et al., 2017) and human blood plasma along with low total and progressive sperm motility (Cito et al., 2020). Likewise, the miR-202-5p is a highly expressed factor in mice testis during the early stages of development that re-surge during its final stages of development (Wainwright et al., 2013), posing to be strongly associated with the development of male gonad. Particularly in zebrafish, miR-202-5p shows high levels of expression in germ cells in the different stages of spermatogenesis, including the formation of spermatogonia and maturation of spermatozoa, thereby reinforcing the existence of a relationship between miR-202-5p and sperm maturation in this species (Jia et al., 2015).

In the present study, we observed the down-regulation of both miR-125a and miR-125b-5p expressions in the sperm cells of *gh* + zebrafish. We also reported in our earlier studies that these members of a miR-125 family were down-regulated when tested in nanotransfected sperm cells of *Bos taurus* shown by the negative changes in sperm kinetics and other parameters, such as its membrane integrity, acrosome reaction, and mitochondrial membrane potential (Domingues et al., 2020). A knockout study of miR-125b5p in mice showed it to cause male infertility (Li et al., 2019), indicating that the negative regulation of this miRNA in *gh* + zebrafish found here may also have deleterious effects on the fertilizing potential of the male F0104 zebrafish strain.

Further, the upregulated miR-146b observed in our study correlated with the previously observed abundant levels in bull testicular tissue (Gao et al., 2020), where its increased expression led to the inhibition of proliferation of germline stem cells and simultaneously promoted their apoptosis. Similarly, a deletion in the chromosomal locus for the miR-200a-5p precursor gene showed to increase the kinetics parameters of spermatozoa and significantly improved the fertilization rate in zebrafish in a different study (Xiong et al., 2018). This effect was caused due to the ectopic expression of miR-200a-5p by reducing expressions of its target genes: *wt1a*, *srd5a2b*, and *amh*, which are known to play an important role in spermatozoa motility.

The miR-184 was reported to be upregulated in the low motility fraction of bull sperms (Capra et al., 2017). In humans, the miR-184 plays an important role in male fertility by regulating genes related to spermatogenesis (Rahbar et al., 2020). Overall, the upregulations of these miRNAs show to have a negative effect on male fertility, suggesting that this may be a key reason for decreased fertility in *gh* + zebrafish.

The remaining differentially expressed miRNAs identified in our studies, such as the miR-22a-5p, miR-126, miR-22a, and miR-221 have also been earlier reported in semen samples of zebrafish and other species (Smorag et al., 2012; Jia et al., 2015; Tao et al., 2018; Alves et al., 2021). However, the functional role of these miRNAs and target mRNAs has not yet been fully elucidated. Further, characterization of these candidate miRNAs influencing the zebrafish spermatozoa characteristics and reproductive success is opportunely needed.

The *in silico* analysis of molecular pathways affected by differentially expressed miRNAs confirmed that the *gh* overexpression may be influencing the gene expressions related to pathways associated with spermatic motility. The FoxO signaling pathway, which includes transcription factors regulating the gene expressions related to apoptosis, cell cycle control, glucose metabolism, and resistance to oxidative stress identified in our study, was one of the main target sites for the down-regulated miRNAs (Wang et al., 2014). Therefore, a reduction in the miRNAs targeting *foxo3a* suggests a positive regulation of this transcription factor, consequently activating the FoxO signaling pathway. In addition, several other genes downstream from the coding region for *foxo3a* transcription factor have also shown to be targeted by the down-regulated miRNAs, further implying their importance in this pathway (by themselves or in association with PI3K/Akt signaling pathway) in spermatic cells of *gh* + zebrafish (Griffeth et al., 2013; Capra et al., 2017; Zhang et al., 2017b; Catalano-Iniesta et al., 2019).

In this study we observed an upregulation of miRNAs that affects genes related to Toll-like receptor signaling pathway in spermatic cells of gh-transgenic zebrafish. As discussed by Navarro-Costa et al. (2020), the functional significance of these immunity-related genes (such as *tlr7*, *tlr8, and traf6*) on the sperm motility process is not fully elucidated. Nevertheless, other study demonstrated that stimulated Toll-like receptor signaling reduce sperm motility and suppress fertilization in human (Fujita et al., 2011); Furthermore, a number of TLRs in testes of yellow catfish were significantly reduced by chloroquine treatment, resulting in an improved sperm motility and fertilization rate (Zhang et al., 2017a). Interestingly, in the present study, the affected main target gene present in TLR signaling pathway was *pik3cb*. As previously demonstrated, introducing a germline point mutation in *pik3cb* gene resulted in oligo- or azoospermia phenotypes in mice (Guillermet-Guibert et al., 2015). Taken together, these data suggest that this molecular pathway is closely related to sperm motility in different species, the result of an energetic trade-off between reproduction and immune responses. The negative regulation of miRNAs affects the genes downstream of the p53 signaling pathway in *gh* + zebrafish spermatozoa, indicating the activation of this pathway. These targets include those that induce cell-cycle arrest and initiate apoptosis by regulating several pro-apoptotic genes in another pathway that may be affected by the down-regulated miRNAs. As previously discussed, some events that occur during apoptosis, such as decreased mitochondrial functionality, spermatic membrane integrity, and DNA integrity, have also been observed in *gh* + zebrafish and appear to be involved in reducing fertilization and hatching frequency in these species (Figueiredo et al., 2013). In addition to the activation of the FoxO signaling pathway; therefore, the miRNAs also favor the expression of these pro-apoptotic genes (Wang et al., 2014).

Further, to comprehensively explore the biomarker potential of these miRNAs, a sensitive, accurate, and cost-efficient miRNA profiling technique is required. Next-generation sequencing (NGS) is emerging as a preferred method for miRNA profiling; as it offers high sensitivity, single-nucleotide resolution, and the possibility to profile a considerable number of samples in parallel (Coenen-Stass et al., 2018). Taking the above advantages into consideration, Vaz et al. (2015) demonstrated miRNA expression levels by miRNA sequencing of adult zebrafish tissues (brain, gut, liver, ovary, testis, eye, and heart) and compared with qPCR results showed good correlation for both known miRNAs and unknown miRNAs categories.

In addition to the above-mentioned study, identification and expression profile of miRNAs in spermatic cells and gonad of distinct species, such as zebrafish (Presslauer et al., 2017), Nile tilapia *Oreochromis niloticus* (Tao et al., 2016), *Bos taurus* (Stowe et al., 2014; Capra et al., 2017), *Sus scrofa* (Godia et al., 2019), and human (Hua et al., 2019; Xu et al., 2020), were done using NGS, without the need for further validation by qPCR.

The *in silico* analysis of pathways affected by the regulatory activity of down-regulated miRNAs identified by our research has risen some significant queries that need to be further examined, most notably the inference of miRNA sequencing data in F0104 males, a suitable translational model for studying the unpredictable collateral effects of excess GH on reproductive traits especially on the pathophysiology of male infertility by unknown, apparent cause. Follow-up studies may include experimental validation of functional interactions of miRNAs-target genes, using methods such as the luciferase assay report, which are undoubtedly required to understand the epigenetic mechanisms by which *gh* overexpression could contribute to a reduced reproductive potential.

In our study, in addition to the epigenetic modulation effect on spermatic cells from *gh* + zebrafish, we show the F0104 transgenic strain overexpressing GH showed a bodyweight 1.2 times greater than that of NT animals, confirming that the increased growth hormone led to stimulation of zebrafish muscle growth. Studies previously carried out using the same transgenic zebrafish strain reported similar muscle growth due to high levels of GH and thereby IGF1 expression, highly affecting the gene expressions essential for increased growth (Rosa et al., 2008; Kuradomi et al., 2011; Figueiredo et al., 2013; Silva et al., 2015; Nornberg et al., 2016).

Previous studies have demonstrated the differential expression of miRNA to be strongly associated with certain male reproductive dysfunctions, especially the movements of spermatozoa. Kinetic parameters are one of the main characteristic features of spermatozoa important for fertilization. A decrease in total motility and duration of spermatic motility observed in *gh* + zebrafish was in accordance with the previous findings in the F0104 strain (Figueiredo et al., 2013). In addition to motility, spermatic cells in *gh* + fish show decreased mitochondrial activity, membrane integrity, and DNA stability as the most probable reasons for reduced fertilization and hatching frequency seen in these fish (Figueiredo et al., 2013). Further, a significant increase in metabolic rate, generation of reactive oxygen species (ROS) (Rosa et al., 2008), and decrease in gene expressions related to the antioxidant defense system (Batista et al., 2014) in *gh* + fish can lead to oxidative stress, that in turn may cause deleterious effects on mitochondrial activity and ATP synthesis thereby reducing the fertilizing potential of spermatozoa.

In summary, our results conclude that *gh* overexpression alters the microRNAome expression profile of spermatic cells in zebrafish. The identified predicted gene targets from the up-and down-regulated miRNAs provide scope for future studies that involve validation of these miRNA-targets interactions to use these candidate miRNAs as fertility biomarkers across species. Further, the *gh*-transgenic zebrafish strain may be used as a suitable model in reproductive translational research.

## Data Availability Statement

The datasets presented in this study can be found in online repositories. The names of the repository/repositories and accession number(s) can be found below: https://www.ncbi.nlm.nih.gov/, SRP318063.

## Ethics Statement

The animal study was reviewed and approved by the Ethics Committee of the Federal University of Rio Grande (FURG), Brazil, under the code 23116.008403/2018–32.

## Author Contributions

VC, WD, LM, and TS conceived and planned the experiments. TS, MK, and LM generated and raised the animals. WD, EB, IA, AV, and CC carried out the laboratorial analyses (spermatic kinetic and molecular biology). GG, CR, and WD contributed to miRNA libraries preparation. LN, AS, and DP contributed to the interpretation of the results. WD took the lead in writing the manuscript. All authors provided critical feedback and helped shape the research, analysis, and manuscript.

## Conflict of Interest

The authors declare that the research was conducted in the absence of any commercial or financial relationships that could be construed as a potential conflict of interest.

## Publisher’s Note

All claims expressed in this article are solely those of the authors and do not necessarily represent those of their affiliated organizations, or those of the publisher, the editors and the reviewers. Any product that may be evaluated in this article, or claim that may be made by its manufacturer, is not guaranteed or endorsed by the publisher.

## Footnotes

1
https://www.zfin.org

2
https://bioinfo5.ugr.es/srnatoolbox

## Supplementary Material

Supplementary Figure 1 Qualitative analysis of RNA quality isolated from zebrafish sperm cells samples. Representative total RNA samples quality parameter analyzed by TapeStation 4200. RNA from sperm cells of *gh*-transgenic (A1, B1, and C1) zebrafish (*Danio rerio*) belonging to the F0104 strain and non-transgenic groups (D1, E1, and F1) are compared with electronic Ladder (EL).
