# Supplementary figures and images for: GH Overexpression Alters Spermatic Cells MicroRNAome Profile in Transgenic Zebrafish
Source: Front Genet. 2021 Sep 8;12:704778. doi: 10.3389/fgene.2021.704778 (PMC8455951; doi:10.3389/fgene.2021.704778)

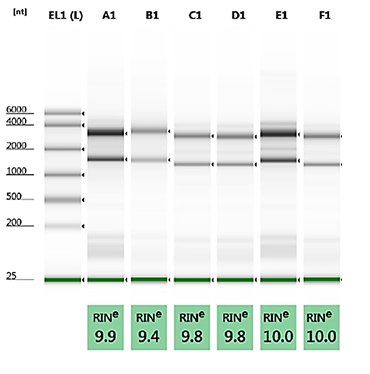

Supplement: Supplementary Figure 1 — Qualitative analysis of RNA quality isolated from zebrafish sperm cells samples. Representative total RNA samples quality parameter analyzed by TapeStation 4200. RNA from sperm cells of gh-transgenic (A1, B1, and C1) zebrafish (Danio rerio) belonging to the F0104 strain and non-transgenic groups (D1, E1, and F1) are compared with electronic Ladder (EL). [file Image_1.TIF]
