# Supplementary material for: GH Overexpression Alters Spermatic Cells MicroRNAome Profile in Transgenic Zebrafish
Source: Front Genet. 2021 Sep 8;12:704778. doi: 10.3389/fgene.2021.704778 (PMC8455951; doi:10.3389/fgene.2021.704778)
Supplement: Supplementary file 2 [file Table_1.DOCX]

**Table S1**. **Raw and statistics data of differentially expressed miRNAs between *gh-*transgenic and non-transgenic zebrafish sperm.**

| **miRNA name** | **RPM** | | | | | | **Fold Change** | **log2 Fold Change** | **p-value** | **padj** | **Regulated** |
| --- | --- | --- | --- | --- | --- | --- | --- | --- | --- | --- | --- |
|  | **NT1** | **NT2** | **NT3** | **GH1** | **GH2** | **GH3** |  |  |  |  |  |
| dre-miR-19d-3p | 40.749 | 71.583 | 12.887 | 1.39E-17 | 1.39E-17 | 1.39E-17 | 0.1106 | -6.781 | 0.0022 | 0.0214 | down |
| dre-miR-126a-5p | 8.449 | 7.234 | 49.374 | 1.39E-17 | 1.39E-17 | 1.39E-17 | 0.127 | -6.538 | 0.0005 | 0.008 | down |
| dre-miR-126b-5p | 8.449 | 7.234 | 49.374 | 1.39E-17 | 1.39E-17 | 1.39E-17 | 0.127 | -6.539 | 0.0005 | 0.008 | down |
| dre-miR-22a-5p | 62.206 | 61.804 | 72.736 | 11.614 | 12.253 | 2.566 | 0.1473 | -2.638 | 0.0001 | 0.0044 | down |
| dre-miR-16c-5p | 71.298 | 137.07 | 106.4 | 25.05 | 19.158 | 37.615 | 0.1604 | -2.491 | 0.0002 | 0.0056 | down |
| dre-miR-20a-5p | 98.133 | 87.484 | 87.674 | 22.352 | 20.466 | 36.912 | 0.1792 | -2.336 | 0.0006 | 0.008 | down |
| dre-miR-126b-3p | 48.978 | 34.013 | 68.931 | 15.23 | 96.603 | 26.526 | 0.1971 | -2.211 | 0.0016 | 0.017 | down |
| dre-miR-107a-3p | 39.964 | 52.614 | 67.919 | 16.119 | 11.007 | 28.044 | 0.2014 | -2.175 | 0.0015 | 0.017 | down |
| dre-miR-93 | 68.008 | 73.639 | 97.709 | 13.368 | 13.742 | 20.115 | 0.2072 | -2.428 | 0.001 | 0.0123 | down |
| dre-miR-2189 | 509.83 | 268.3 | 472.02 | 115.18 | 148.78 | 54.618 | 0.2566 | -1.919 | 0.0004 | 0.0069 | down |
| dre-miR-202-5p | 3727.6 | 3654.2 | 2300.6 | 885.72 | 885.85 | 811.36 | 0.267 | -1.905 | 1.50E-05 | 0.0007 | down |
| dre-miR-221-3p | 622.4 | 562.63 | 742.68 | 256.34 | 140.96 | 121.69 | 0.2704 | -1.866 | 0.0003 | 0.0064 | down |
| dre-miR-125a | 1716.1 | 2185.1 | 2678.6 | 1048.3 | 193.16 | 738.51 | 0.3012 | -1.735 | 0.0021 | 0.0214 | down |
| dre-miR-125b-5p | 2369.6 | 2344.3 | 3561.7 | 1136.7 | 456.9 | 935.25 | 0.3058 | -1.713 | 0.0004 | 0.0069 | down |
| dre-miR-126a-3p | 1935.3 | 1355.7 | 1570.3 | 698.22 | 672.62 | 671.76 | 0.4205 | -1.251 | 0.0025 | 0.0237 | down |
| dre-miR-30c-5p | 1033.3 | 1844.4 | 1311.7 | 674.13 | 535.98 | 590.88 | 0.4303 | -1.217 | 0.0046 | 0.0391 | down |
| dre-miR-146b | 275.39 | 245.55 | 136.39 | 951.76 | 1435.2 | 906.04 | 4.991 | 23.284 | 1.91E-06 | 0.0001 | up |
| dre-miR-200a-5p | 45.196 | 46.908 | 15.262 | 15.984 | 63.609 | 50.262 | 6.378 | 30.631 | 0.0037 | 0.0328 | up |
| dre-miR-146a | 198.21 | 251.64 | 97.449 | 807.08 | 2182.6 | 805.21 | 6.901 | 27.984 | 1.14E-06 | 0.0001 | up |
| dre-miR-726 | 66.781 | 17.786 | 38.255 | 39.251 | 78.83 | 5.91 | 8.309 | 33.843 | 0.0008 | 0.0107 | up |
| dre-miR-184 | 0.7082 | 63.687 | 0.8965 | 29.363 | 152.24 | 68.107 | 17.44 | 46.059 | 8.45E-05 | 0.0031 | up |
| dre-miR-738 | 27.169 | 23.167 | 41.594 | 1396.6 | 1082.5 | 104.58 | 70.58 | 6.268 | 4.10E-09 | 7.63E-07 | up |
